# Metalloprotease OMA1 Fine-tunes Mitochondrial Bioenergetic Function and Respiratory Supercomplex Stability

**DOI:** 10.1038/srep13989

**Published:** 2015-09-14

**Authors:** Iryna Bohovych, Mario R. Fernandez, Jennifer J. Rahn, Krista D. Stackley, Jennifer E. Bestman, Annadurai Anandhan, Rodrigo Franco, Steven M. Claypool, Robert E. Lewis, Sherine S. L. Chan, Oleh Khalimonchuk

**Affiliations:** 1 Department of Biochemistry; 2 Redox Biology Center and; 3 School of Veterinary Medicine and Biomedical Sciences, University of Nebraska-Lincoln, Lincoln NE, 68588, USA; 4 Eppley Institute for Research in Cancer, University of Nebraska Medical Center, Omaha, NE 68198, USA; 5 Department of Drug Discovery and Biomedical Sciences, Medical University of South Carolina, Charleston, SC 29425, USA; 6 Department of Physiology, Johns Hopkins School of Medicine, Baltimore, MD 21205, USA

## Abstract

Mitochondria are involved in key cellular functions including energy production, metabolic homeostasis, and apoptosis. Normal mitochondrial function is preserved by several interrelated mechanisms. One mechanism – intramitochondrial quality control (IMQC) – is represented by conserved proteases distributed across mitochondrial compartments. Many aspects and physiological roles of IMQC components remain unclear. Here, we show that the IMQC protease Oma1 is required for the stability of the respiratory supercomplexes and thus balanced and tunable bioenergetic function. Loss of Oma1 activity leads to a specific destabilization of respiratory supercomplexes and consequently to unbalanced respiration and progressive respiratory decline in yeast. Similarly, experiments in cultured Oma1-deficient mouse embryonic fibroblasts link together impeded supercomplex stability and inability to maintain proper respiration under conditions that require maximal bioenergetic output. Finally, transient knockdown of OMA1 in zebrafish leads to impeded bioenergetics and morphological defects of the heart and eyes. Together, our biochemical and genetic studies in yeast, zebrafish and mammalian cells identify a novel and conserved physiological role for Oma1 protease in fine-tuning of respiratory function. We suggest that this unexpected physiological role is important for cellular bioenergetic plasticity and may contribute to Oma1-associated disease phenotypes in humans.

Generation of ATP through the tricarboxylic acid (TCA) cycle and oxidative phosphorylation (OXPHOS) is one of the vital mitochondrial functions. Electrons derived from the TCA cycle are fed into the OXPHOS system consisting of the electron transport chain (ETC) complexes (Complex I–IV) and ATP synthase (Complex V). Electron flux via the ETC units generates a proton gradient, which is subsequently used by Complex V to generate ATP^1^. Transfer of reactive electrons via the multiprotein, membrane-embedded ETC complexes is almost unavoidably linked to formation of superoxide radicals generated by incomplete reduction of molecular oxygen - the final acceptor of electrons funneled through ETC^2^. These byproducts of respiration can be further converted into other potent radicals and are commonly known as reactive oxygen species (ROS). Perturbations in the electron transport can further increase ROS production by ETC^2^. Accumulating or persisting ROS can damage biomolecules located in the vicinity of the ETC and cause mitochondrial dysfunction and disease^3^,^4^.

Individual ETC complexes are organized into so-called respiratory chain supercomplexes (RSCs) that localize to specific invaginations of the inner mitochondrial membrane (IMM) termed cristae^5^. The RSCs are believed to optimize electron channeling through ETC units and permit a robust use of various available substrates/electron donors, thereby assuring optimal and adaptable OXPHOS function^6^,^7^. It has also been postulated that RSCs help to enhance electron flux and minimize loss of the electrons - and thus ROS production - by ETC^8^,^9^. Relatively little is known about factors that promote and regulate formation and stability of RSCs. In yeast, stability of supercomplexes appears to be impacted by several factors: 1) phospholipid composition of IMM^10^,^11^,^12^; 2) subunits of cytochrome *c* oxidase (CcO, Complex IV)^13^,^14^; 3) ATP/ADP exchanger Aac2^15^,^16^; and 4) IMM-anchored proteins Rcf1 and Rcf2^9^,^13^,^17^. Similar factors seem to contribute to RSCs’ stability in mammals. A recent study identified an important role of cristae and cristae-remodeling factors like IMM GTPase OPA1 in assembly and stability of mammalian RSCs^18^.

A subset of conserved proteases is involved in maintenance of protein homeostasis in the IMM^4^,^19^. IMM-anchored *m*-AAA, *i*-AAA and Oma1 metalloproteases have been implicated in proteolytic control of ETC assembly/stability^4^,^20^,^21^,^22^. These enzymes mediate proteolysis and removal of misfolded or unassembled OXPHOS subunits, thereby preventing malformation of dysfunctional ETC units, which upon accumulation can affect electron flux, promote excessive ROS production and negatively impact IMM integrity^4^. While Oma1 is known to mediate degradation of hypo-hemylated Cox1 CcO subunit in yeast^20^,^23^, its primary role in metazoans appears to be proteolytic processing of OPA1^24^,^25^,^26^,^27^. However, the diversity of phenotypes associated with Oma1 loss^28^,^29^ suggests that the protease might be playing additional roles in mitochondrial physiology.

Here, we describe a novel role of Oma1, wherein this ATP-independent protease is involved in stabilization of RSCs at different stages of life. Yeast cells deficient in Oma1 display decreased supercomplex stability, unbalanced respiration and progressive respiratory decline during aging. Studies in zebrafish and mammalian cell culture models further support our conclusions and suggest a conserved physiological role of Oma1 in fine-tuning of respiratory function in metazoans through control of RSCs stability/performance. Our data provide novel insights into the physiological significance of Oma1 in cellular health and protection against aging-associated mitochondrial degeneration.

## Results

### Progressive respiratory decline in the aging oma1Δ mutant.

**Figure 1:**
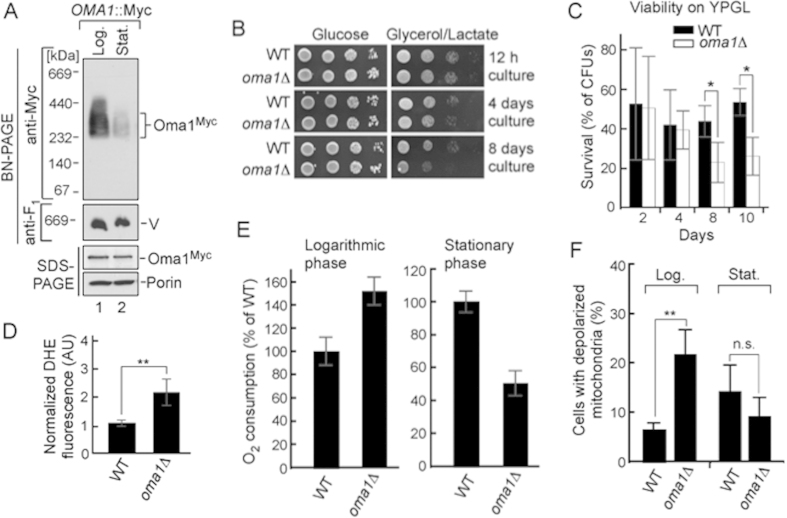
Deletion of Oma1 leads to progressive mitochondrial dysfunction. (**A**) Electrophoretic analyses of mitochondria from wild-type (WT) strain with Oma1-13xMyc chromosomal tag isolated at exponential (12 h post-inoculation, A_600_ of 0.8; Log.) and stationary (48 h post-inoculation, A_600_ of 8.0; Stat.) phases of growth. Mitochondria (70 μg) were solubilized with 1.5% digitonin and subjected to BN-PAGE; 20 μg of mitochondria were used for SDS-PAGE. Oma1 was detected by immunoblotting with anti-Myc. Monomeric form of Complex V (V; BN-PAGE loading control) visualized with anti-F_1_ serum; porin was SDS-PAGE loading control. Source data (full-length blots) are available online in Supplementary information. (**B**) Respiratory growth of WT and *oma1*Δ strains. Synchronized cells were cultured in YPD medium for 0.5 (12 hours), 4 and 8 days at 28 °C and spotted onto YPD (Glucose) or YPGL (Glycerol/Lactate) plates. Pictures taken after 2 (YPD) or 4 (YPGL) days of growth at 28 °C. (**C**) WT and *oma1*Δ strains were grown in YPD medium for indicated number of days, diluted to 600 cells and plated on YPGL plates. Following a 4-days incubation at 28 °C, number of colony forming units was determined (n = 3 biologically independent experiments). The values were normalized to number of colonies that each strain formed when cultured on glucose-supplemented plates. (**D**) Endogenous superoxide levels in WT and *oma1*Δ cells. Log-phase cells stained with O_2_^.−^-specific dye dihydroethidium (DHE) were analyzed by flow cytometry (n = 4). (**E**) Oxygen consumption of synchronized WT and *oma1*Δ cells at log (A_600_ of 0.8) and stationary (A_600_ of 8.0) stages of growth; n = 3 independent cultures per each strain. (**F**) Mitochondrial membrane potential of WT and *oma1*Δ strains during log and stationary growth, assessed by flow cytometry analysis of JC-1-stained cells (n = 3). Data represent mean values ± S.D. **p < 0.01, *p < 0.05, n.s. =  non-significant (*t*-test).

Our group and others have previously reported that loss of Oma1 function in yeast does not result in any appreciable phenotype under normal growth conditions^23^,^26^,^28^. However, Oma1 proteolytic activity is important for cellular tolerance to various conditions of mitochondrial stress^28^. It was shown that Oma1 function is critical for stationary phase survival of yeast cells deficient in *m*-AAA or *i*-AAA proteases, as well as manganese superoxide dismutase Sod2^26^,^28^. We also noticed that activity, but not abundance of the oxidative damage-susceptible enzyme aconitase tended to decrease in Oma1-deficient cultures maintained into stationary phase (Ref. 28 and data not shown). Consistent with the role of Oma1 in mitochondrial health in stationary phase, we observed destabilization of the Oma1 oligomeric complex from these cells by BN-PAGE, as compared to the oligomer in mitochondria from log phase cells (Fig. 1A). Steady-state levels of the protease were similar in each case. Changes in Oma1 oligomer stability upon BN-PAGE reflect the enzyme’s activation^28^ and indicate that the protease is more active in stationary cells. We hypothesized that Oma1 deficit may lead to age-associated decline of mitochondrial health. Cell survival in stationary phase heavily depends on ATP generated via mitochondrial respiration^30^. We thus examined time-dependent maintenance of respiratory competence by wild type (WT) and *oma1*Δ cells. Respective strains were cultured for a period of 12 hours (log phase), 4 days (stationary phase) or 8 days (late stationary phase) and tested for their ability to propagate on plates containing a non-fermentable glycerol/lactate mix. Respiratory growth of the *oma1*Δ mutant was comparable to WT at both 12 hours and 4 days of culture, but at day 8 of culture we observed significant decrease in the ability of *oma1*Δ mutant to propagate on glycerol/lactate plates (Fig. 1B). Glucose growth of WT and *oma1*Δ cells was normal at all culturing periods tested. This result suggests specific time-dependent impairment of respiratory function in *oma1*Δ strain. To substantiate our conclusion, we quantitatively assessed the ability of WT and *oma1*Δ strains to form viable colonies on glycerol/lactate medium upon prolonged culturing. No significant difference in respiratory survival of the strains was observed after 2 or 4 days of culture. In contrast, *oma1*Δ mutant exhibited substantial decline in survival on glycerol/lactate medium after 8 days of culture (Fig. 1C). The survival of *oma1*Δ cells on the plates containing glucose or galactose was not affected (Supplementary Fig. S1A and data not shown). Exponentially growing *oma1*Δ cells displayed about two-fold increase in endogenous superoxide (as indicated by staining with O_2_^−^-specific dye dihydroethidium) than WT strain (Fig. 1D), thereby implicating ROS as a likely contributor to observed respiratory decline. We then tested mitochondrial respiration of WT and *oma1*Δ strains at different stages of growth in galactose-containing medium. Galactose is a fermentable carbon source, which however, does not repress mitochondrial function. Surprisingly, the *oma1*Δ mutant displayed significantly higher rate of oxygen consumption in the log phase (12 h of culture), as compared to WT (Fig. 1E). However, in stationary phase (4 days of culture) *oma1*Δ cells displayed marked decrease in oxygen consumption (Fig. 1E), indicating mitochondrial impairment. In line with respiratory imbalance, the *oma1*Δ mutant exhibited alterations in mitochondrial membrane potential, as indicated by higher percentage of depolarized cells during exponential growth (Fig. 1F). These data demonstrate that cells lacking Oma1 have unbalanced respiration and are prone to a gradual decline of respiratory function with age.

### Loss of Oma1 affects the stability of RSCs without impeding individual ETC units.

**Figure 2:**
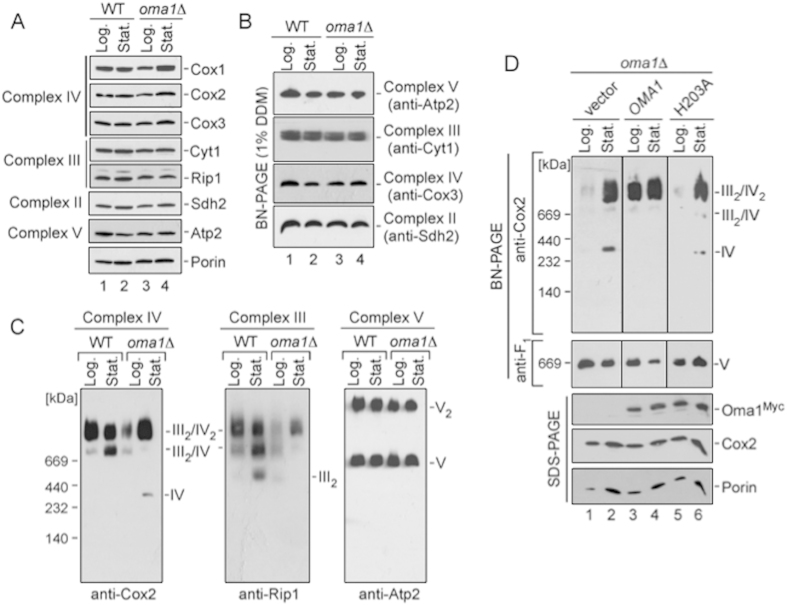
Respiratory supercomplexes are impaired in Oma1-deficient yeast cells. (**A**) Steady-state levels of the Cox1-Cox3 subunits of CcO (Complex IV), Cyt1 and Rip1 subunits of *bc*_1_cytochrome *c* reductase (Complex III), Sdh2 subunit of succinate dehydrogenase (Complex II), and Atp2 subunit of F_1_F_O_ ATP synthase (Complex V), and porin were assessed by immunobloting of mitochondria (20 μg) from WT and *oma1*Δ cells. (**B**) BN-PAGE of individual ETC complexes from log and stationary phase WT and *oma1*Δ cells. Mitochondria (40 μg) were solubilized with 1% dodecyl maltoside (DDM). The complexes were visualized by blotting with indicated antibodies. (**C**) BN-PAGE of the above mitochondria lysed with 1.5% digitonin. (**D**) *oma1*Δ cells bearing vector, Myc-tagged Oma1 or its H203A variant were grown in synethetic galactose medium and used for mitochondrial isolation. Mitochondria (70 μg) were analyzed by BN-PAGE as in C. Another 20 μg of mitochondria were used for SDS-PAGE. Source data (full-length blots) are available online in Supplementary information.

To gain more insights into the role of Oma1 in respiration, we conducted a thorough analysis of the ETC function in *oma1*Δ mutant at both log and stationary phases of growth. Steady-state levels of key subunits of OXPHOS complexes were unaffected by loss of Oma1 in both galactose and glucose cultures (Fig. 2A). Similarly, mitochondrial translation rates and stability of newly synthesized Complex IV (Cox1, Cox2, Cox3), Complex III (Cytochrome *b*) and Complex V (Atp6) subunits in *oma1*Δ mutant were comparable to WT (Supplementary Fig. S1B). BN-PAGE analysis of individual OXPHOS units (obtained through lysis of the respective mitochondria with 1% dodecylmaltoside) also did not reveal significant differences in abundance or stability of these complexes in the *oma1*Δ strain (Fig. 2B). Equally, specific enzymatic activities of CcO (Complex IV), *bc*_1_ cytochrome *c* reductase (Complex III) and succinate dehydrogenase (Complex II) were similar in both WT and *oma1*Δ mitochondria (Supplementary Fig. S1C-S1E). These data indicate that unbalanced respiration seen in the *oma1*Δ mutant is most likely not due to an impairment of individual OXPHOS units. Next, we examined if observed oxygen consumption imbalance in *oma1*Δ, cells may be due to compromised integrity of Complex III/Complex IV RSCs – a condition that is known to affect respiratory rates^31^. Synchronized WT and *oma1*Δ strains were cultured in galactose-containing media to promote mitochondrial function and mitochondria were isolated from respective cells harvested at either log or stationary phases. The organelles were lysed in mild non-ionic detergent digitonin to preserve RSCs. Consistent with previous report^9^, BN-PAGE of WT lysates showed that in the log phase both Complex III (anti-Rip1 immunoblot) and Complex IV (anti-Cox2 immunoblot) associate into III_2_/IV_2_ tetramer and, to a smaller extent, into III_2_/IV trimer. Abundance of the latter RSC increased and some dimeric Complex III was also seen in the stationary phase in WT mitochondria (Fig. 2C). In contrast, analysis of RSCs in *oma1*Δ mutant revealed marked reduction in III_2_/IV_2_ supercomplex in log stage and III_2_/IV supercomplex in stationary stage, respectively. In addition, we observed accumulation of free Complex IV in stationary *oma1*Δ cells (Fig. 2C), indicating that RSCs are likely less stable under BN-PAGE conditions if Oma1 is lost. Migration patterns of Complex V (monomer and dimer) were comparable between WT and *oma1*Δ cells. To confirm that the observed RSCs’ stability defect arises from Oma1 deficit, we examined the distribution of III/IV supercomplexes in *oma1*Δ cells expressing WT protease or its catalytic H203A mutant^28^. As shown in Fig. 2D, despite the fact that either Oma1 variant was present at similar levels, only the expression of WT protease but not its catalytic mutant, stabilized the III/IV RSCs. Together, our data demonstrate that loss of Oma1 function impairs the stability of RSCs. Oma1 does not appear to be a stoichiometric component of RSCs as no interaction between the protease and subunits of ETC complexes was observed (Ref. 20 and data not shown).

### *OMA1* genetically interacts with RSCs-stabilizing factors.

**Figure 3:**
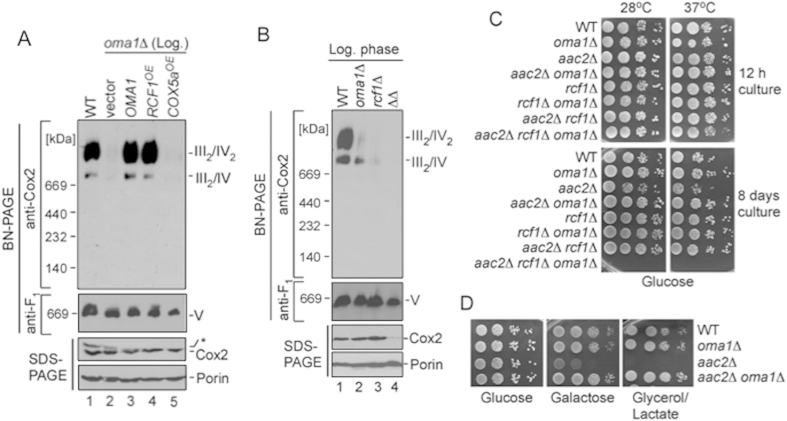
*OMA1* genetically interacts with supercomplex-stabilizing factors. (**A**) BN- and SDS-PAGE analysis of mitochondria from log-phase *oma1*Δ cells expressing *OMA1*, overexpressing *RCF1* or *COX5a*. Source data (full-length blots) are available online in Supplementary information. (**B**) Mitochondria from log-phase wild-type (WT), *oma1*Δ *rcf1*Δ and *rcf1*Δ *oma1*Δ cells were analyzed by native (70 μg of mitochondria) or denaturing (20 μg of mitochondria) PAGE. (**C**) Indicated strains were cultured in YPD medium for 0.5 (12 hours) and 8 days at 28 °C, spotted onto YPD (Glucose) plates and incubated at 28 °C or 37 °C. Pictures were taken after 2 days of growth. (**D**) The indicated strains were dropped onto YPD (Glucose), YPGal (Galactose) and YPGL (Glycerol/Lactate) plates and incubated at 28 °C.

We sought to identify factors that may contribute to RSCs destabilization seen in Oma1-deficient cells. Factors known to impact RSCs’ stability include phospholipid composition of the IMM and several IMM-anchored proteins like Rcf1/HIG1 and Rcf2/HIG2^9^,^13^,^17^, ATP/ADP carrier Aac2^15^,^16^ and Complex III/IV interface-mediating protein Cox13^31^. The impediment in RSCs’ stability seen in *oma1*Δ mutant was not due to changes in mitochondrial phospholipid content or altered phospholipid/protein ratio (Supplementary Fig. S2A, B). We thus focused on analysis of genetic interactions of *OMA1* with genes encoding factors that are known to influence the stability of III/IV RSCs. First, we examined the ability of aforementioned molecules to stabilize labile RSCs in *oma1*Δ mutant. Overexpression of *RCF1*, but not genes encoding other RSCs-stabilizing factors like CcO subunit Cox5a, stabilized the III/IV supercomplexes in mitochondria isolated from log-phase *oma1*Δ cells (Fig. 3A and not shown). Reciprocally, loss of Rcf1 in *oma1*Δ background further compromised stability of RSCs in this strain (Fig. 3B). Such destabilization is likely due to affected stability of the core subunits of Complex IV, as indicated by anti-Cox2 immunoblot (Fig. 3A and Supplementary Fig. S2C). We then tested the genetic relationship between Oma1 and RSCs-stabilizing factors in greater detail. Because some single deletion mutants in question are unable to grow efficiently on non-fermentable media, we employed a previously reported approach, whereby stationary phase survival of double deletion strains on glucose-containing plates is used as readout for respiratory capacity^9^,^30^. We constructed a series of strains lacking Oma1, Rcf1, Cox13, or Aac2 in different combinations. Respective deletion mutants were tested for survival on glucose plates at 28 °C and 37 °C. When grown to log stage, all strains displayed normal growth at tested conditions (Fig. 3C and data not shown). Similarly, when tested at day 6 of stationary phase, *oma1*Δ, *rcf1*Δ and *cox13*Δ mutants as well as *aac2*Δ *oma1*Δ, *rcf1*Δ *aac2*Δ, *cox13*Δ *oma1*Δ and *rcf1*Δ *oma1*Δ *cox13*Δ strains grew normally at both 28 °C and 37 °C. Growth of *aac2*Δ mutant was slightly delayed at 37 °C. In contrast, stationary phase-grown *rcf1*Δ *oma1*Δ *aac2*Δ triple mutant showed profound survival defect at either temperature (Fig. 3C). Similar results were obtained when cells were cultured on galactose plates (not shown).

Our observation that the *aac2*Δ mutant, but not the *aac2*Δ *oma1*Δ strain exhibited 37 °C growth defect suggests that loss of *OMA1* may have an alleviating effect in the *aac2*Δ background. We thus examined the ability of these strains to propagate on fermentative (glucose), non-repressive (galactose) and respiratory (glycerol/lactate) media. Consistent with previous report^32^, the *aac2*Δ strain grew poorly on galactose medium and was unable to form viable colonies on YPGL plates. Conversely, the *oma1*Δ *aac2*Δ mutant displayed robust growth on YPGL medium, indicating that loss of Oma1 can alleviate Aac2 deficiency (Fig. 3D). Notwithstanding positive genetic effect, RSCs in the *aac2*Δ *oma1*Δ mutant remained unstable (not shown). As expected, deletion of *RCF1* in *aac2*Δ *oma1*Δ strain ablated the suppressive effect of Oma1 loss (not shown). These data demonstrate genetic interaction between *RCF1, AAC2* and *OMA1* suggesting that products of these genes may act either in parallel or at different steps to promote RSCs stability. However, because Aac2 plays several important roles in mitochondrial physiology^33^, observed genetic interaction may not be solely attributed to the molecule’s role in RSCs stabilization and will be a subject of future analyses.

### Loss of Oma1 leads to developmental defects in zebrafish model.

**Figure 4:**
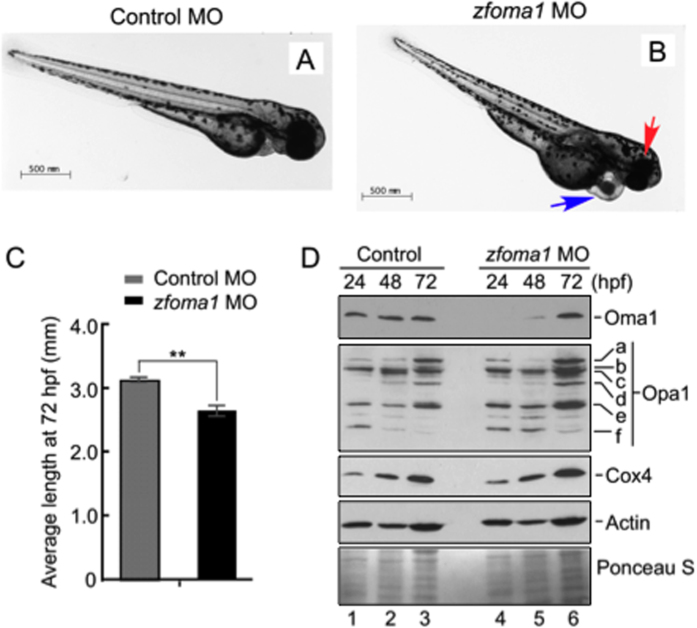
Depletion of Oma1 in fish affects development. (**A,B**) Representative images of larval zebrafish at 72 hours post fertilization (hpf) injected with either a standard control MO (**A**) or a *zfoma1* MO (**B**). Fish injected with *zfoma1* MO have smaller heads and eyes (red arrow) and defects in heart morphology (blue arrow). Bar, 500 μm. (**C**) Body length of control and *zfoma1* morphants at 72 hpf. Average standard length was plotted ± S.E.M. (n = 6–13, **p < 0.01 by *t*-test). (**D**) Immunoblot of protein extracts from injected fish (yolk removed) at 24, 48 and 72 hpf. Expression of Oma1, Opa1 isoforms (a–f) and Cox4 (mitochondrial abundance marker) was analyzed with respective antibodies. Actin and Ponceau S staining were loading controls. Source data (full-length blots) are available online in Supplementary information.

To better understand the physiological role of Oma1 in context of metazoan animal development, we examined effects of Oma1 depletion in a *Danio rerio* vertebrate model. Four-cell stage fish embryos from the same breeding pairs were injected with either control, or *zfoma1*-specific translation blocking morpholinos (MO). Initial analysis of embryos at 72 hours post fertilization (hpf) revealed that unlike control morphants, Oma1-depleted fish presented with pericardial edema, smaller eyes and shorter body length (Fig. 4A–C). Immunoblotting of protein extracts from de-yolked embryos with antibodies against human OMA1 (also recognizes the fish homolog) confirmed an efficient depletion of the protease (Fig. 4D). The highest reduction of Oma1 levels was observed at 24 and 48 hpf. At 72 hpf, intensity of the Oma1 band in the *zfoma1* morphant was increased, likely due to decreased MO effectiveness. Steady-state levels of Cox4, a key CcO subunit, and Actin were comparable in each case (Fig. 4D). We next examined abundance and distribution of the known OMA1 substrate, OPA1. Consistent with a previous report^34^, multiple isoforms of the protein were detected. Distribution of Opa1 isoforms was comparable between control and Oma1 morphants at all times tested (Fig. 4D). Interestingly, the abundance of Opa1-specific bands was slightly increased in *zfoma1*-treated embryos. Together, these data demonstrate that Oma1 depletion in fish leads to a series of organ developmental defects characteristic of mitochondrial dysfunction. Observed defects do not appear to be due to instability or changes in basal processing of Opa1, or CcO subunits.

### Phenotypic and bioenergetics analysis of *zfoma1* morpholino-treated zebrafish embryos.

**Figure 5:**
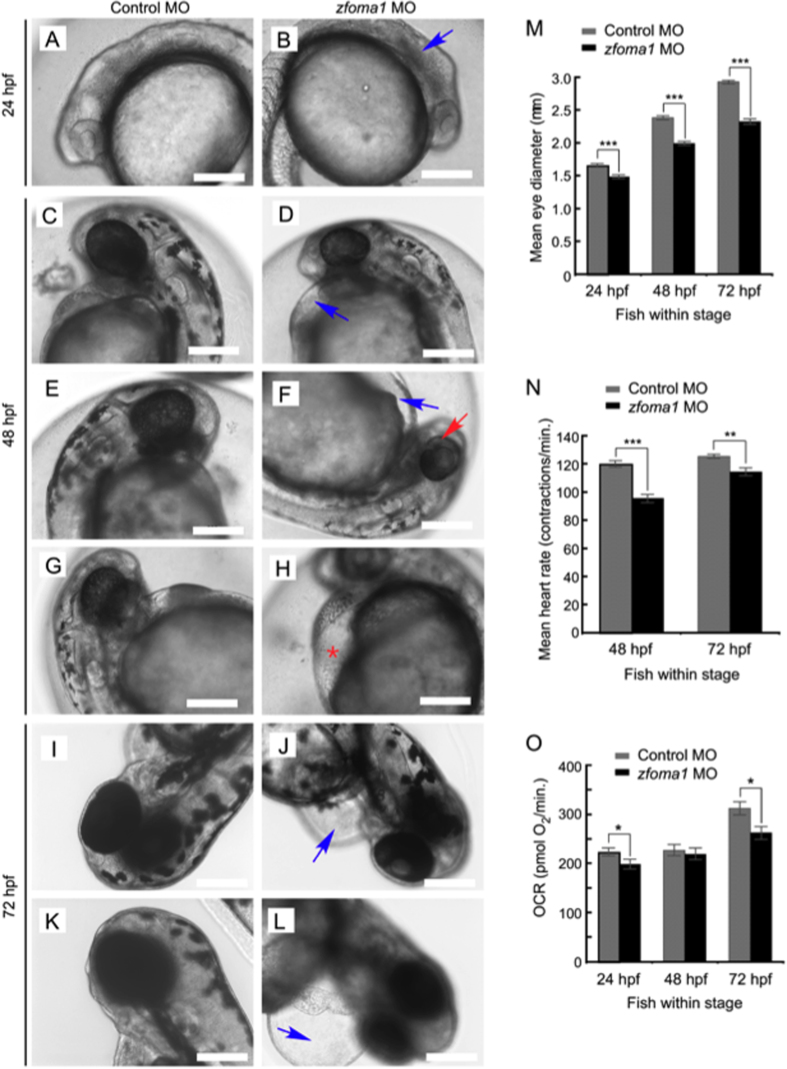
Depletion of Oma1 in fish results in specific developmental abnormalities. (**A–L**) At 24 hpf, *zfoma1* morphants (B) lack definition of brain structures (blue arrow) compared to control MO fish (**A**). At 48 hpf, several abnormalities are observed in Oma1 MO-injected embryos (**D**,**F**,**H**). The embryos had smaller heads and eyes and often exhibited pericardial edema (blue arrow, **D**,**F**) that sometimes resulted in visible erythrocyte accumulation in the yolk sinus area (red asterisk, **H**). Pigmentation in the eye was partially complete compared to controls (red arrow, **F**). At 72 hpf, pericardial edema was extensive (blue arrow, **J**,**L**) and hearts were largely unlooped. Scale bars, 200 μm. (**M**) Eye size in control and *zfoma1* morphants at 24, 48 and 72 hpf (n = 9–12). (**N**) Heart contraction rates (measured as beats per minute) in control versus Oma1-depleted fish at 48 and 72 hpf (n = 30). (**O**) *In vivo* respiration in control and Oma1 MO-treated fish embryos at 24, 48 and 72 hpf (n=10). Data are shown as mean ± S.E.M.; *p < 0.05, **p < 0.01, ***p < 0.001 *zfoma1* vs. control MO (*t*-test).

Because the most efficient depletion of Oma1 is seen at 24–72 hpf, we sought to analyze phenotypic consequences of *zfoma1* MO treatment at these stages of life. At 24 hpf, *zfoma1* morphants did not exhibit significant differences in cardiac morphology as compared to control embryos. This is likely due to the fact that zebrafish hearts are still developing at this stage. However, we observed substantial graininess of the neural tube in *zfoma1* MO fish, which reflects brain abnormality (Fig. 5A,B). Such abnormality was not pronounced at 48 hpf. However, we noticed that Oma1-depleted fish exhibited significantly smaller and slightly sectored eyes (Fig. 5C–H). In addition, at both 48 and 72 hpf, *zfoma1* MO-treated embryos displayed pericardial edema (Fig. 5C–L) that was often accompanied by accumulation of red blood cells in the yolk sinus area (Fig. 5H, red asterisk), suggesting impeded blood circulation. Quantitative assessment of eye sizes in control and Oma1 morphants revealed that the latter animals had significantly smaller eyes at all stages tested (Fig. 5M). Likewise, heart contractility of 48 and 72 hpf *zfoma1* MO fish - as reflected by decreased heart rate - was lower when compared to control (Fig. 5N).

The Oma1 morphants exhibit phenotypes known to associate with bioenergetic defects^34^,^35^. We thus interrogated rates of oxygen consumption (OCR) - a proxy for mitochondrial respiratory function - by control and Oma1-deficient embryos. Basal OCR for respective morphants at 24–72 hpf were determined using XF24 extracellular flux analyzer^36^. At 24 and 72 hpf, respiration was substantially decreased in Oma1-depleted embryos, when compared to control (Fig. 5O). Of note, we observed that at 48 hpf, OCRs of control and Oma1 morphants were not significantly different, likely due to a compensatory mechanism. These data indicate that eye and cardiovascular developmental defects seen in *zfoma1* MO fish arise from an impaired bioenergetic function.

### Impaired bioenergetics capacity in *oma1* ^−/−^ MEFs.

**Figure 6:**
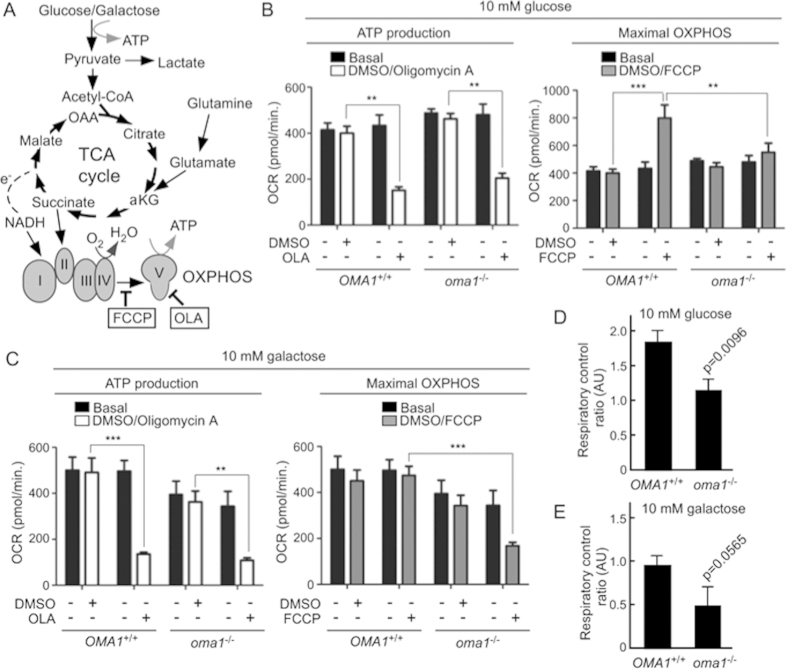
Impaired bioenergetic function in *oma1*^−/−^ MEFs. (**A**) Schematic of cellular energy-converting pathways and inhibitors used to profile MEFs’ bioenergetics. FCCP, carbonyl cyanide 4-(trifluoromethoxy)phenylhydrazone; OLA, oligomycin A. (**B,C)** Oxygen consumption rates (OCR) in wild type (*OMA1*^+/+^) and *oma1*^−/−^ MEFs under basal, OLA- and FCCP-stimulated conditions. Cells were cultured in the media containing 10 mM glucose (B) or 10 mM galactose (C). Data are shown as mean ± S.E.M. (n = 3 biological replicates); *p < 0.05, **p < 0.01, ***p < 0.001 (unpaired *t*-test). (**D,E**) Respiratory control ratios (a ratio between FCCP-stimulated OCR and basal OCR) in *OMA1*^+/+^ and *oma*^−/−^ cells cultured in 10 mM glucose (**D**) or 10 mM galactose (**E**). Error bars indicate S.D.; *p* values are relative to control.

Based on the above observations, we hypothesized that developmental defects seen in Oma1 morphants can be - at least in part - due to mitochondrial deficits caused by destabilized RSCs. Because an in-depth analysis of mitochondrial function in fish embryos is challenging, we instead decided to address this question by using *oma1*^−*/*−^ mouse embryonic fibroblasts (MEFs). A previous study^29^ reported that cells derived from *oma1*^−*/*−^ mice do not exhibit significant respiratory deviations upon standard culturing (in presence of glucose and glutamine). Under these conditions, fibroblasts derive ATP from both aerobic glycolysis and glutamine oxidation^37^ (Fig. 6A). However, as suggested by our analyses in yeast, consequences of Oma1 deficit may become apparent only when cells primarily rely on OXPHOS. We thus measured OCR by WT and *oma1*^−*/*−^ MEFs cultured in media containing 10 mM glucose or 10 mM galactose. Either condition is known to force cells to switch towards glutaminolysis and mitochondrial respiration^37^,^38^. No significant differences between basal OCR and ATP production rates (measured as OCR due to inhibition of ATP synthase with oligomycin) in WT and *oma1*^−*/*−^ MEFs were seen under each condition tested (Fig. 6B,C). Nevertheless, when MEFs were incubated with the drug FCCP - to uncouple ETC from the proton gradient and thus determine maximal OXPHOS activity - we observed that *oma1*^−*/*−^ MEFs were unable to increase their OCR in response to the treatment (Fig. 6B,C). Of note, FCCP treatments of *oma1*^−*/*−^ MEFs cultured in 10 mM galactose were unable to restore OCR to basal levels as observed for WT MEFs. Moreover, the observation that FCCP did not increase OCR above basal levels in WT MEFs (in comparison to WT MEFs cultured in 10 mM glucose) indicates that 10 mM galactose-grown MEFs have already maximized OXPHOS. Together, these data suggest that Oma1-deficient mitochondria are unable to perform at peak respiratory capacity, when maximal flux via the ETC is allowed. We also noticed that when cultured in 10 mM glucose-containing medium, *oma1*^−*/*−^ MEFs exhibited higher extracellular acidification rate (ECAR) upon oligomycin or FCCP-treatments (Supplementary Fig. S3A, S3B), reflecting enhanced compensatory aerobic glycolysis. Incubation of cells with glycolysis inhibitor 2-deoxyglucose decreased ECAR to levels seen with WT fibroblasts, indicating that glycolytic reserve of *oma1*^−*/*−^ MEFs was not altered (Supplementary Fig. S3C). To substantiate our findings, we determined the ratio between FCCP-uncoupled OCR and basal OCR, known as respiratory control ratio (RCR) - an established measure of mitochondrial efficiency^39^. RCR was significantly lower for *oma1*^−*/*−^ MEFs cultured in medium containing 10mM glucose compared to WT cells (Fig. 6D). A similar pattern was observed for galactose-grown MEFs (Fig. 6E). Importantly, these findings parallel bioenergetic defects seen in *zfoma1* MO-treated fish embryos.

### Mammalian OMA1 is required for RSCs stability.

**Figure 7:**
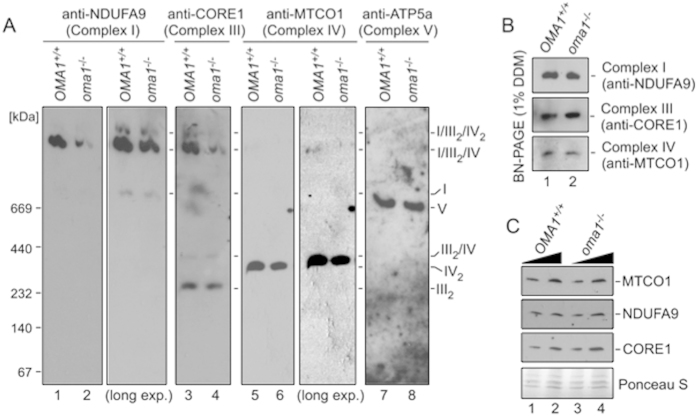
Loss of OMA1 impairs mammalian RSCs. (**A**) BN-PAGE of mitochondria from wild type (*OMA1*^+/+^) and *oma1*^−/−^ MEFs. Mitochondria (80 μg) were solubilized with 2% digitonin. Protein complexes were visualized with antibodies to NDUFA9 (Complex I), CORE1 (Complex III), MTCO1 (Complex IV) and ATP5A (Complex V). (**B**) BN-PAGE of WT and *oma1*^−/−^ mitochondrial lysates. Mitochondria (40 μg) were solubilized with 1% dodecyl maltoside (DDM). Individual ETC complexes were visualized by immunoblotting with indicated antibodies. (**C**) Steady-state levels of the indicated subunits of ETC complexes in WT and *oma1*^−/−^ mitochondria. Ten and 15 μg of mitochondria were analyzed by SDS-PAGE. Source data (full-length blots) for key panels of this figure are available online in Supplementary information.

We next tested if the inability to maximize ETC performance seen in *oma1*^−*/*−^ MEFs might arise from destabilized RSCs. BN-PAGE of mitochondrial lysates from WT and *oma1*^−*/*−^ MEFs revealed that the abundance of the I/III_2_/IV_2_ and I/III_2_/IV RSCs was decreased in the latter cells (Fig. 7A). This attenuation appears to be related to a depletion or destabilization of RSCs that contain Complexes I, III and IV; assemblies that lack these complexes (Complex V) remained unperturbed in *oma1*^−*/*−^ MEFs. At the same time, levels of individual Complexes I, III, IV (Fig. 7B), and steady-state levels of representative subunits (Fig. 7C) were largely unchanged. These data indicate that the role of Oma1 in stabilization of RSCs observed in the yeast model is conserved in higher eukaryotes. Bioenergetic deficits observed in *oma1*^−*/*−^ MEFs and zebrafish model are likely consequences of an unbalanced ETC due to impeded stability of RSCs. The molecular bases of Oma1-mediated RSCs’ stabilization in metazoans will be a focus of future studies.

## Discussion

Conserved metalloprotease Oma1 was shown to mediate stress-triggered proteolytic processing of IMM-anchored proteins in both yeast and mammals^24^,^25^,^26^,^27^,^28^,^40^. However, reported stress conditions used to mimic mitochondrial dysfunction and trigger Oma1 activation are often extreme and borderline to non-physiological. While useful for obtaining initial mechanistic insights, this approach leaves many aspects of Oma1′s physiological significance unaddressed. Here, we conducted experiments aiming to unveil additional physiological roles of Oma1. We show herein that Oma1 activity is required for stability of RSCs in both yeast and metazoans. Such stabilization appears to be critical for optimal ETC function and respiration at maximal capacity. Several lines of evidence support these conclusions.

First, stationary yeast cells deficient in Oma1 exhibit progressive respiratory decline. Although this finding *per se* provides only indirect evidence, it is further strengthened by observed imbalance in oxygen consumption displayed by *oma1*Δ mutant in both log and stationary phase of growth. Mitochondrial respiration is enhanced and is critical for survival of yeast cells in stationary phase^30^. It is plausible that accumulating respiratory deficit will lead to a growth defect on non-fermentable media. Noteworthy, observed decrease in oxygen consumption by stationary phase *oma1*Δ strain is seen prior to any respiratory growth defects displayed by these cells. Activation-associated destabilization of the Oma1 complex by BN-PAGE observed in stationary WT cells also indicates the importance of Oma1 for respiratory survival during aging. Curiously, a similar respiratory imbalance pattern has been previously reported in mutants defective in nutrient-sensing TOR signaling as well as yeast cells subjected to dietary restriction regimen^41^. It will be interesting to test whether there is a link between Oma1′s function and TOR signaling. The above findings are paralleled by the results of immunoblot analyses of *oma1*Δ mitochondria from log versus stationary cells. The *oma1*Δ mutant does not display any significant impediment in biogenesis, stability, specific enzymatic activity, or abundance of individual ETC complexes or their core (and presumably supernumerary) subunits. However, BN-PAGE analyses of digitonin extracts suggest that RSCs are less stable in the absence of Oma1. Importantly, similar destabilization is observed in mitochondria isolated from *oma1*^−/−^ MEFs. These data demonstrate evolutionary conservation of Oma1′s role in the stabilization of RSCs. The mechanism of Oma1-mediated RSCs stabilization remains to be clarified. It does not seem to involve phospholipids or changes in the IMM’s lipid to protein ratios. Likewise, direct physical association of Oma1 with RSCs seems unlikely^20^. The mechanism clearly involves the enzyme’s proteolytic function, as the expression of catalytically inactive Oma1 does not confer RSCs’ stability.

Second, our study reveals genetic interaction between Oma1 and Rcf1 - a factor, which was shown previously to mediate the stability of RSCs^9^,^13^,^17^. Moreover, *OMA1* displays strong genetic interaction with *AAC2*, another molecule implicated in stabilization of RSCs^15^,^16^. Loss of Oma1 in Aac2 deletion mutant results in a robust synthetic rescue effect and restores respiratory competence of the *aac2*Δ strain. Interestingly, this genetic effect is not associated with stabilization of RSCs. Because Aac2 plays other (distinct from RSCs stabilization) important roles in mitochondrial physiology^32^,^33^, the observed genetic interaction may not be fully related to RSCs’ stability and requires additional investigation. Nonetheless, 1) the fact that rescue effect seen in *aac2*Δ *oma1*Δ strain is ameliorated by depletion of *RCF1*; and 2) strong synthetic interaction between these three genes in stationary phase survival growth assays, are consistent with the postulated role of Oma1 in RSCs stabilization. Of note, we did not observe strong synthetic interaction between *RCF1* and *AAC2* reported previously^9^. This is likely due to differences in genetic background of strains used in these analyses.

Third, our studies in MEFs demonstrate that loss of Oma1 in mammalian cells appears to have similar outcomes. In agreement with previous report^29^, under standard cultivation conditions, basal OCRs of *oma1*^−/−^ MEFs are similar to those of WT cells. This is probably due to the ability of MEFs to generate ATP from both aerobic glycolysis and mitochondrial glutamine oxidation^37^. However, when *oma1*^−/−^ cells are forced to rely primarily on mitochondrial function by lowering glucose in the medium or replacing it with galactose, the bioenergetic deficit becomes evident. The most striking difference is seen upon uncoupling of cells with FCCP – the condition that maximizes ETC function. Consistent with bioenergetic impediment, this condition significantly enhances aerobic glycolysis in *oma1*^−/−^ but not in WT MEFs cultured in low glucose. One explanation is that upon uncoupling, Oma1-mediated cleavage of L-OPA1 – an important event necessary for cristae remodeling^24^,^27^,^29^,^42^ – may be also required for subsequent OXPHOS “re-tuning”. Inability to remodel mitochondrial cristae via proteolysis of OPA1 under these conditions could be the reason for the observed effects. Indeed, IMM cristae and OPA1 were shown recently as important mediators of RSCs formation and respiratory efficiency^18^,^43^. On the other hand, preservation of long OPA1 isoform and thus robust cristae ultrastructure is viewed as a benefit and was shown to be protective against mitochondrial damage^27^,^29^,^44^. Perhaps the role of Oma1 in RSCs stability is not restricted to OPA1 processing and may involve a different mechanism. Consistent with this hypothesis is our observation that Oma1-mediated stabilization of RSCs also occurs in yeast even though the fungal OPA1 homolog, Mgm1, is not a substrate of yeast Oma1^45^,^46^,^47^. Similarly, bioenergetic defects seen in Oma1-depleted fish embryos do not seem to arise primarily from impaired OPA1 processing. Future analyses will address the question of how condition-induced Oma1-mediated processing of OPA1 impacts the stability of respiratory supercomplexes in mammalian cells.

Finally, results of our experiments in a zebrafish model underscore the physiological significance of Oma1 in supporting proper respiratory function during embryogenesis. Oma1-deficient fish exhibit developmental abnormalities, which are hallmarks of impeded energy metabolism and mitochondrial disease^34^,^35^,^48^. Consistent with the idea that bioenergetic deficit is driving observed developmental defects; *zfoma1* morphants display significantly decreased OCR. This impediment is particularly pronounced at 72 hpf stage wherein fish embryos become more dependent on respiration^36^.

In conclusion, our studies in yeast, zebrafish and mammalian cells suggest a novel and conserved physiological role for Oma1, whereby this ATP-independent protease is involved in stabilization of RSCs and fine-tuning of respiratory function in eukaryotes during various stages of life. Our data provide important insights into physiological significance of Oma1 in cellular health and protection against aging-associated mitochondrial degeneration. We anticipate that these findings might enable new beneficial approaches to enhance mitochondrial health in aging cellular populations.

## Methods

### Yeast Strains, Vectors and Media

This study used *Saccharomyces cerevisiae* strains isogenic to the W303-1B (MATα *ade2-1 his3-1*,*15 leu2-3*,*112 trp1-1 ura3-1*) genetic background. The strains used are listed in Supplementary Table 1. Genetic manipulations were conducted as described previously^28^. Yeast strains were cultured in standard YP or SC medium supplemented with appropriate nutrients and either 2% glucose, 2% galactose or 2% glycerol/lactate as a sole carbon source. Where indicated, yeast strains were transformed with pRS415-Oma1-Myc-6xHis, pRS415-Oma1-Myc-6xHis H203A^28^, pRS426-Rcf1^14^ and pRS423-Cox5a^49^ plasmids.

### Yeast Assays

The respiratory competence of indicated yeast strains was assessed by growth tests on YP plates containing 2% glucose, 2% galactose or 2% glycerol/2% lactate as a carbon source. Synchronized yeast cells were grown for indicated period of time at 28 °C in liquid medium containing glucose, adjusted to A_600_ of 1.0 and serially diluted and spotted onto the respective plates. The plates were incubated at 28 °C for 2 days (YPD and YPGal) or 4–6 days (YPGL). Alternatively, aliquots of the yeast culture were diluted to 600 cells and plated onto YPD or YPGL plates. The colony forming units were scored following 2 (YPD) or 4 (YPGL) days incubation at 28^o^C. The oxygen consumption of yeast strains in question was determined using an Oxygraph system (Hansatech Instruments) according to the manufacturer’s instructions. The rate of oxygen consumption (presented as % of wild type) was calculated from the linear response. *In vivo* labeling of mitochondrial translation products was done as described previously^49^.

### Flow Cytometry

The fluorescence-assisted sorting was done with BD FACSCanto^TM^ II system (Becton Dickinson). The measurements were performed using 1 ml of exponentially grown yeast cells diluted in 900 μl of pre-warmed PBS (28^o^C). Accumulation of superoxide was measured using DHE (Cayman Chemical) as described previously^41^. Briefly, 1 ml the yeast culture was incubated with 5 μg DHE for 10 min at 30 °C. Fluorescence intensity was measured in the FL2 channel (PE). Analysis of mitochondrial membrane potential was performed with JC-1 (Enzo Life Sciences) in the FL1 (FITC) and FL2 (PE) channels according to the manufacturer’s instructions. Prior to the analysis, 1 ml of yeast cells in PBS was incubated with 0.5 μg JC-1 for 15 min at 37 °C. The percentage of depolarized cells was calculated from the ratio of FITC-positive cells to PE-positive cells. The data were collected, analyzed and plotted using BD FACSDiva software v6.1.1 (Becton Dickinson).

### Zebrafish Maintenance and Care

Wild-type zebrafish (AB strain) were obtained from the Zebrafish International Resource Center (supported by NIH grant P40RR012546). Fish were maintained and crossed according to standard methods^50^. All animal studies were approved by the MUSC Institutional Animal Care and Use Committee (AR #2850) and performed in accordance with the guidelines. Fertilized eggs were collected and placed in E3 embryo medium (5 mM NaCl, 0.17 mM KCl, 0.33 mM CaCl_2_, 0.33 mM MgSO_4_), and maintained in an incubator set at 28.5 °C with a 14/10 h light/dark cycle. Embryos were staged using the criteria of Kimmel *et al.*^51^. The standard control morpholino (Control; 5′- CCTCTTACCTCAGTTACAATTTATA-3′) and Oma1 translation-blocking morpholino (OMA1 TB; 5′-TCTTCTGCAAGGGCTTCAGCACCAT-3′) were designed with and obtained from Gene Tools, LLC. Morpholinos were diluted in sterile distilled H_2_O to a stock concentration of 6 mM and further diluted to 1 mM. Working solutions of phenol red were prepared by diluting neat phenol red (Sigma) 1:10 with 1x sterile Danieau solution (58 mM NaCl, 0.7 mM KCl, 0.4 mM MgSO_4_, 0.6 mM Ca(NO_3_)_2_, 5 mM HEPES, pH 7.2). Morpholinos were titrated to determine the amount of morpholino required for phenotypic differences without toxicity. 3 nL Oma1 TB or control morpholino was injected into the 1–4 cell stage embryo (<0.5 hpf). Final concentration of morpholino in injection solution was 0.2 mM for TB or control by dilution with phenol red. Embryos/larvae were viewed using a Zeiss Axio Observer A.1 inverted microscope with 2.5× and 10× objectives.

Heart rates were measured by counting the number of atrial contractions in 30 seconds in embryos or larvae under 100× magnification. Zebrafish eye size was determined using Axiovision software (Zeiss). Individual images of embryos or larvae were examined and a free-hand circle drawn around the circumference of each eye. The program calculated the area of each circle and the mean values were plotted. To determine body length of larvae, a line was drawn from the tip of the nose to the caudal peduncle of each larvae and the measurement collected and converted to mm based on a micrometer scale. For protein extraction, 20 embryos were pooled for each time point, de-yolked, flash-frozen and stored at −80 °C.

### Metabolic Assays

The oxygen consumption rates (OCR) or extracellular acidification rates (ECAR) were determined using XF24-3 Analyzer (Seahorse Biosciences). 5 × 10^4^ WT or *oma1*^−/−^ MEFs were incubated in DMEM containing 4 mM L-glutamine, 1% NEAA, 1% penicillin-streptomycin and either 10 mM glucose or 10 mM galactose for 72 h prior to plating cells in 24-well XF microplate. After an overnight incubation in microplate, MEFs were switched over to bicarbonate-free low buffer DMEM containing either 10 mM glucose or 10 mM galactose. OCR and ECAR measurements were performed prior to and after consecutive injections of 1 μM Oligomycin A and 1 μM FCCP and 1 mM 2-deoxy-D-Glucose (2-DG) utilizing a 4–2-2 minute mix-wait-measure cycle. Measurements for wells treated with vehicle only were done in duplicate, whereas measurements for drug-treated wells were carried out in quadruplicate.

### Isolation of Mitochondria and Assays

Yeast mitochondria were isolated according to published protocol^52^. For isolation of mammalian mitochondria, 4 × 10^7^ MEFs were cultured in DMEM/F-12 media supplemented with 10% (v/v) fetal bovine serum, penicillin (200 U/ml), streptomycin (200 μg/ml), 2 mM L-glutamine, and 200 μM β-mercaptoethanol. MEFs were released with trypsin, washed twice with PBS and resuspended in homogenization buffer (10 mM HEPES, pH 7.5, 250 mM sucrose, 10 mM MgCl_2_, 10 mM KCl, 1 mM EDTA and 2 mM PMSF). The cells were then disrupted and mitochondria isolated as described^9^. BN-PAGE analyses were performed as described^53^. Unless specified, all gels have been run under the same experimental conditions. Specific activities of respiratory chain enzymes were determined according to published procedures^54^.

### Phospholipid Analyses

Phospholipid extraction, staining, and quantitation were performed essentially as described^15^. In brief, phospholipids were extracted from 1 mg of mitochondria with chloroform:methanol, loaded onto ADAMANT TLC plates (Machery-Nagel), and resolved once in chloroform:ethanol: H_2_O:triethylamine (30:35:7:35). Phospholipids were visualized using a 1.3% molybdenum blue spray reagent (Sigma). Phosphate concentration of extracted lipids was performed as described^55^.

### Immunoblotting

Proteins or protein complexes were detected with indicated primary antibodies and visualized with horseradish peroxidase-coupled secondary antibodies and chemiluminescent reagents (Millipore). The following primary antibodies were used: α-Myc (#11667203001; Roche Diagnostics); α-Cox1 (ab110270), α-Cox2 (ab110271), α-Cox3 (ab110259), α-MTCO1 (ab14705), α-NDUFA9 (ab14713), α-COXIV (ab16056), α-CORE1 (ab110252) and α-ATP5A (ab14748) from Abcam; α-OMA1 (ARP52818_P050; Aviva Systems Biology); α-Porin (#459500; Invitrogen); α-OPA1 (NB110-55290; Novus Biologicals); and α-β-Actin (A2228; Sigma). The polyclonal antibodies against Atp2 (anti-F_1_) subunit of complex V, Cyt1 and Rip1 subunits of complex III, and Sdh2 subunit of complex II were kind gifts from Drs. Alex Tzagoloff (Columbia University), Carla Koehler (UCLA) and Dennis Winge (University of Utah), respectively. Obtained images were scanned and cropped for the figures with cropping lines indicated. Images of full-length blots for key data of each figure are available in the Supplementary information.

### Statistical Analyses

Statistical analyses were conducted using Microsoft Excel. At least three independent replicates have been obtained for each presented experiment. Data are presented as means ± standard error of the mean (S.E.M.) or ± standard deviation (S.D.). Statistical significance was determined using Student’s *t*-test and considered significant at *p *≤ 0.05.

## Additional Information

**How to cite this article**: Bohovych, I. *et al.* Metalloprotease OMA1 Fine-tunes Mitochondrial Bioenergetic Function and Respiratory Supercomplex Stability. *Sci. Rep.* **5**, 13989; doi: 10.1038/srep13989 (2015).

## Supplementary Material

Supplementary Information
