# Supplementary material for: Metalloprotease OMA1 Fine-tunes Mitochondrial Bioenergetic Function and Respiratory Supercomplex Stability
Source: Sci Rep. 2015 Sep 14;5:13989. doi: 10.1038/srep13989 (PMC4568518; doi:10.1038/srep13989)
Supplement: Supplementary Information [file srep13989-s1.pdf]

**SUPPLEMENTARY INFORMATION**

# Metalloprotease OMA1 Fine-tunes Mitochondrial Bioenergetic Function and Respiratory Supercomplex Stability

Iryna Bohovych[1,2], Mario R. Fernandez[4], Jennifer J. Rahn[5], Krista D. Stackley[5], Jennifer E. Bestman[5], Annadurai Anandhan[2,3], Rodrigo Franco[2,3], Steven M. Claypool[6], Robert E. Lewis[4], Sherine S. L. Chan[5] & Oleh Khalimonchuk[1,2*]

*[1]Department of Biochemistry, [2]Redox Biology Center and [3]School of Veterinary Medicine and Biomedical Sciences, University of Nebraska-Lincoln, Lincoln NE, 68588, USA. [4]Eppley Institute for Research in Cancer, University of Nebraska Medical Center, Omaha, NE 68198, USA. [5]Department of Drug Discovery and Biomedical Sciences, Medical University of South Carolina, Charleston, SC 29425, USA. [6]Department of Physiology, Johns Hopkins School of Medicine, Baltimore, MD 21205, USA.*

*Address correspondence to: Oleh Khalimonchuk, Department of Biochemistry and Redox Biology Center, University of Nebraska-Lincoln, 1901 Vine St. N230 BEAD, Lincoln, NE 69588, USA. Tel: (402) 472-8060, Fax: (402) 472-7842. Email: okhalimonchuk2@unl.edu

## SUPPLEMENTAL DATA

### Supplemental Tables

**Table S1**. Genotypes and sources of yeast strains used in this study.

| Strain | Genotype | Reference |
|---|---|---|
| W303-1B | *MATα ade2-1 his3-1,15 leu2-3,112 trp1-1 ura3-1* [*rho*$^+$] | A. Barrientos |
| *oma1Δ* | *MATα ade2-1 his3-1,15 leu2-3,112 trp1-1 ura3-1 oma1Δ:: TRP1 [rho$^+$]* | Ref. 28 |
| *rcf1Δ* | *MATa ade2-1 his3-1,15 leu2-3,112 trp1Δ ura3-1 rcf1Δ::URA3MX* [*rho*$^+$] | This study |
| *rcf1Δ oma1Δ* | *MATa ade2-1 his3-1,15 leu2-3,112 trp1Δ ura3-1 rcf1-Δ oma1Δ::URA3MX [rho$^+$]* | This study |
| *cox13Δ* | *MATa ade2-1 his3-1,15 leu2-3,112 trp1Δ ura3-1 cox13Δ::URA3MX* [*rho*$^+$] | This study |
| *cox13Δ oma1Δ* | *MATa ade2-1 his3-1,15 leu2-3,112 trp1Δ ura3-1 cox13-Δ oma1Δ::URA3MX [rho$^+$]* | This study |
| *aac2Δ* | *MATa ade2-1 his3-1,15 leu2-3,112 trp1Δ ura3-1 aac2Δ::URA3MX* [*rho*$^+$] | This study |
| *aac2Δ oma1Δ* | *MATa ade2-1 his3-1,15 leu2-3,112 trp1Δ ura3-1 aac2-Δ oma1Δ::URA3MX [rho$^+$]* | This study |
| *aac2Δ rcf1Δ* | *MATa ade2-1 his3-1,15 leu2-3,112 trp1Δ ura3-1 aac2-Δ rcf1Δ::URA3MX [rho$^+$]* | This study |
| *rcf1Δ oma1Δ cox13Δ* | *MATa ade2-1 his3-1,15 leu2-3,112 trp1Δ ura3-1 rcf1-Δ oma1-Δ cox13Δ::URA3MX [rho$^+$]* | This study |
| *rcf1Δ oma1Δ aac2Δ* | *MATa ade2-1 his3-1,15 leu2-3,112 trp1Δ ura3-1 rcf1-Δ oma1-Δ aac2Δ::URA3MX [rho$^+$]* | This study |

### Supplemental Figure Legends

**Figure S1, related to Figures 1 and 2. Fermentative growth and expression and activities of OXPHOS complexes in *oma1Δ* mutant.** (A) WT and *oma1Δ* strains were cultured in YPD medium for indicated number of days, diluted to 600 cells and plated on YPGal plates. After 4-days incubation at 28$^{o}$C, the number of colony forming units was assessed for each strain (n=3 biological replicates). Error bars indicate S.D.

(B) *In vivo* pulse-chase labeling of mitochondrial translation products in log-phase wild type (WT) and *oma1Δ* strains. The cells were pulsed for 15 min with [$^{35}$S]-methionine at 30$^{o}$C. Following a 60 min chase at 30$^{o}$C, the samples were separated by SDS-PAGE and analyzed by autoradiography.

(C-E) Specific enzymatic activities of individual respiratory complexes IV (CcO, panel C), III ($bc_1$, panel D) and II (SDH, panel E) in WT and *oma1*Δ mitochondria isolated from the respective strains at exponential or stationary stages of growth. Error bars indicate S.D. (n=3).

**Figure S2, related to Figure 2. Analysis of phospholipid content in Oma1-deficient cells at different stages of growth.** (A) Phospholipds were extracted from 1 mg of mitochondria isolated from indicated strains at log- or stationary-phases of growth and analyzed by thin layer chromatography. Separated phospholipids were visualized with molybdenum blue. Mitochondria from cardiolipin-deficient *crd1*Δ strain served as a control. PC, phosphatidylcholine; PI, phosphatidylinositol; PS, phosphatidylserine; PE, phosphatidylethanolamine; PA, phosphatidic acid; CL, cardiolipin.

(B) Phospholipid to protein ratios in mitochondria derived from indicated strains. Data are mean ± S.E.M. (n=3).

(C) Steady-state levels of CcO subunits Cox1, Cox2 and Cox3 in mitochondria (20 μg) from indicated strains. The outer mitochondrial protein porin was used a loading control.

**Figure S3, related to Figure 6. Effects of *OMA1* deletion on cellular energy metabolism of mouse embryonic fibroblasts.** (A and B) Extracellular acidification rates (ECAR) reflecting aerobic glycolysis in wild type (*OMA1*$^{+/+}$) and *oma1*$^{-/-}$ mouse embryonic fibroblasts under basal, oligomycin A- (OLA, panel A) or carbonyl cyanide 4-(trifluoromethoxy)phenylhydrazone- (FCCP, panlel B) stimulated conditions. Cells were cultured in media containing 10 mM glucose. Data are presented as mean ±S.E.M. (n=3; ***p*<0.01, ****p*<0.001 by unpaired *t*-test).

(C) ECAR in *OMA1*$^{+/+}$ and *oma1*$^{-/-}$ fibroblasts cultured in medium containing 10 mM glucose or its non-metabolizing analog 2-deoxy-D-Glucose (2-DG).

**Figure S4.** Source data for Figure 1A.

**Figure S5.** Source data for Figure 2A-D.

**Figure S6.** Source data for Figure 3A.

**Figure S7.** Source data for Figure 4D.

**Figure S8.** Source data for key panels of Figure 3A-C.

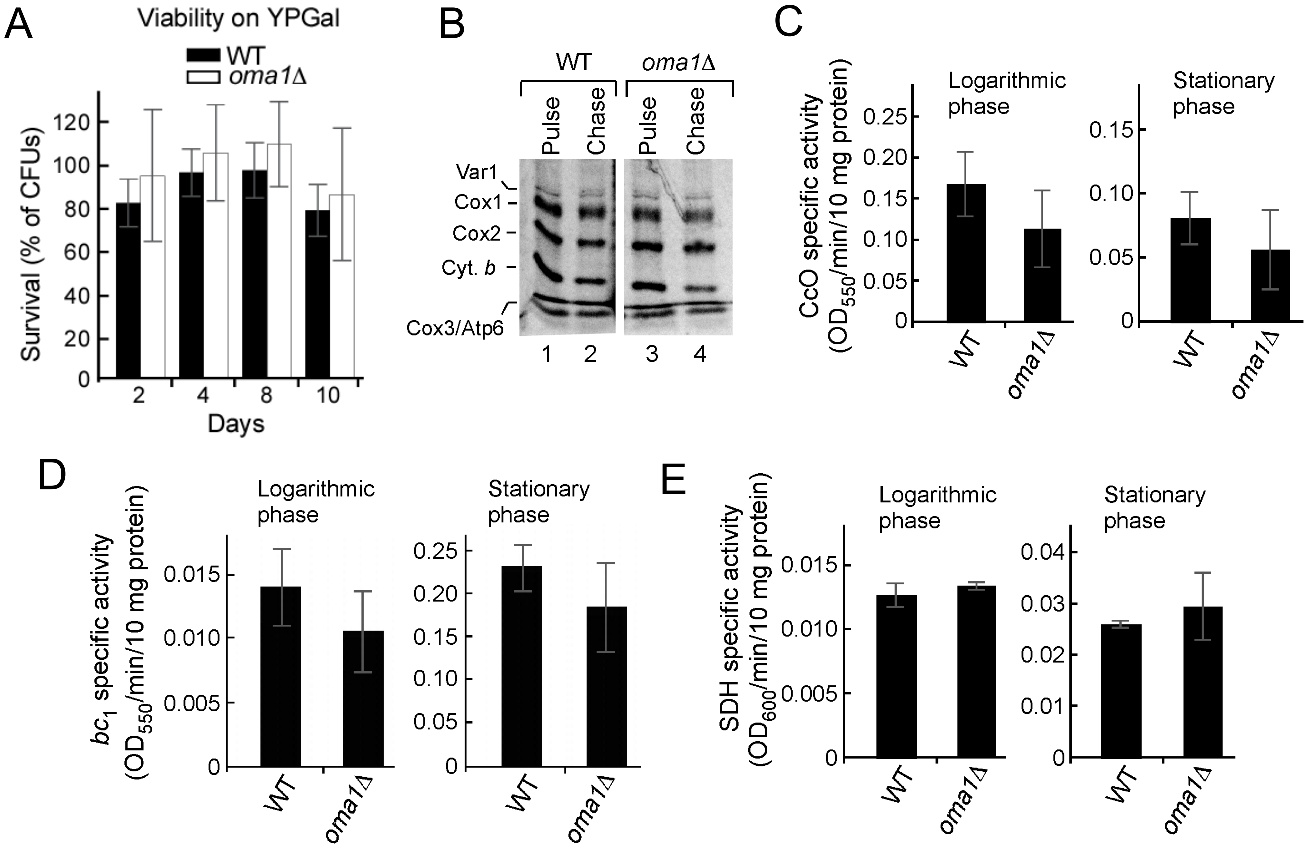


Bohovych, Figure S1

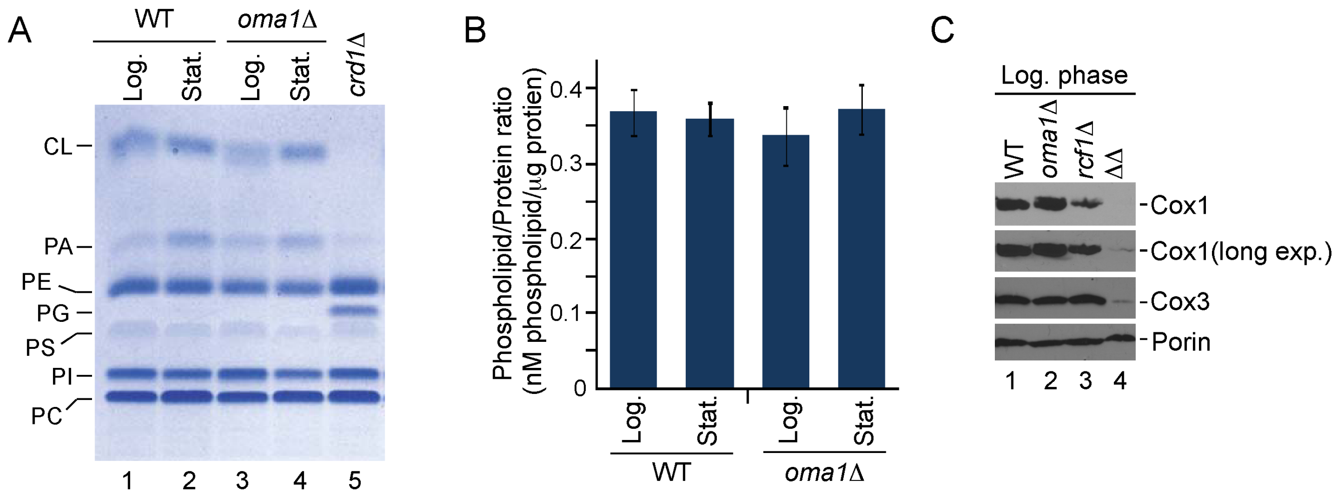


Bohovych, Figure S2

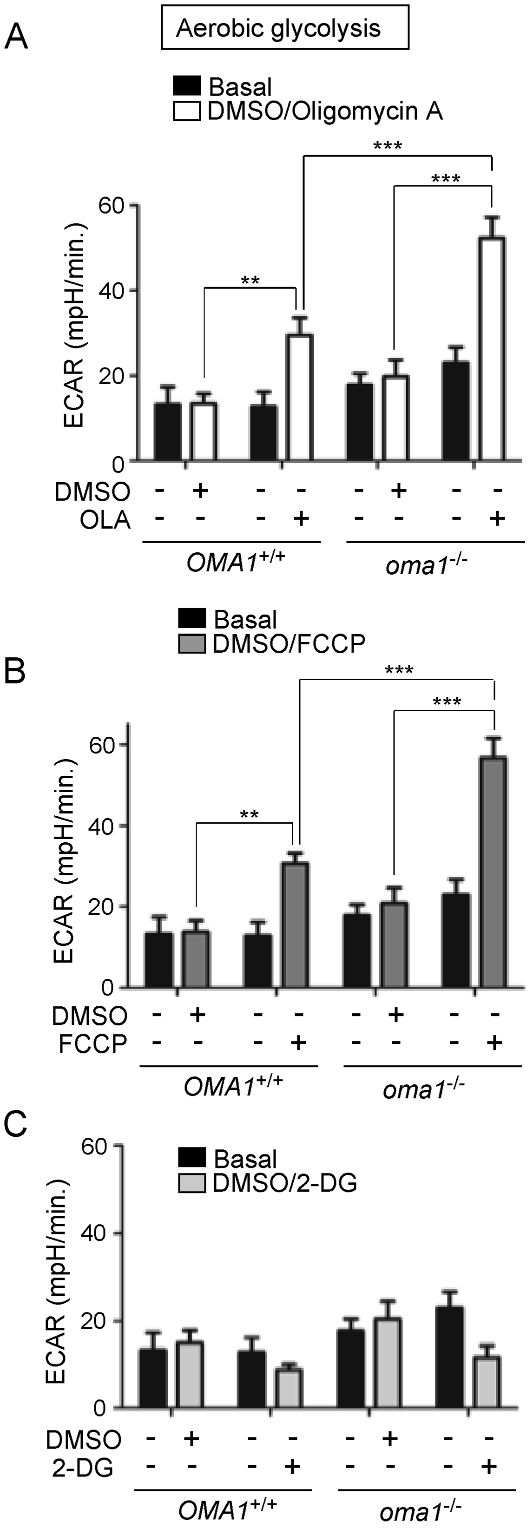


Bohovych, Figure S3

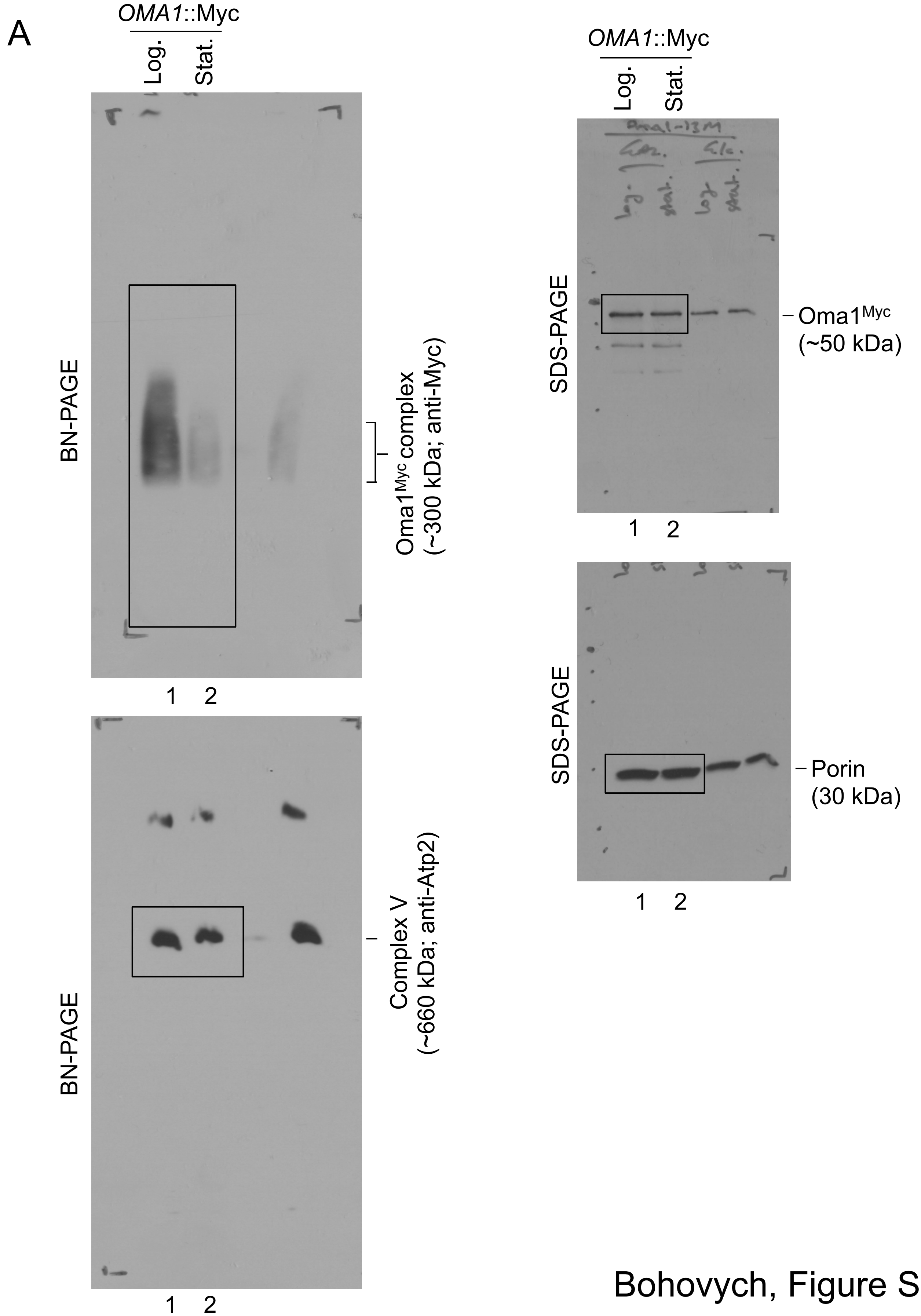


Bohovych, Figure S4

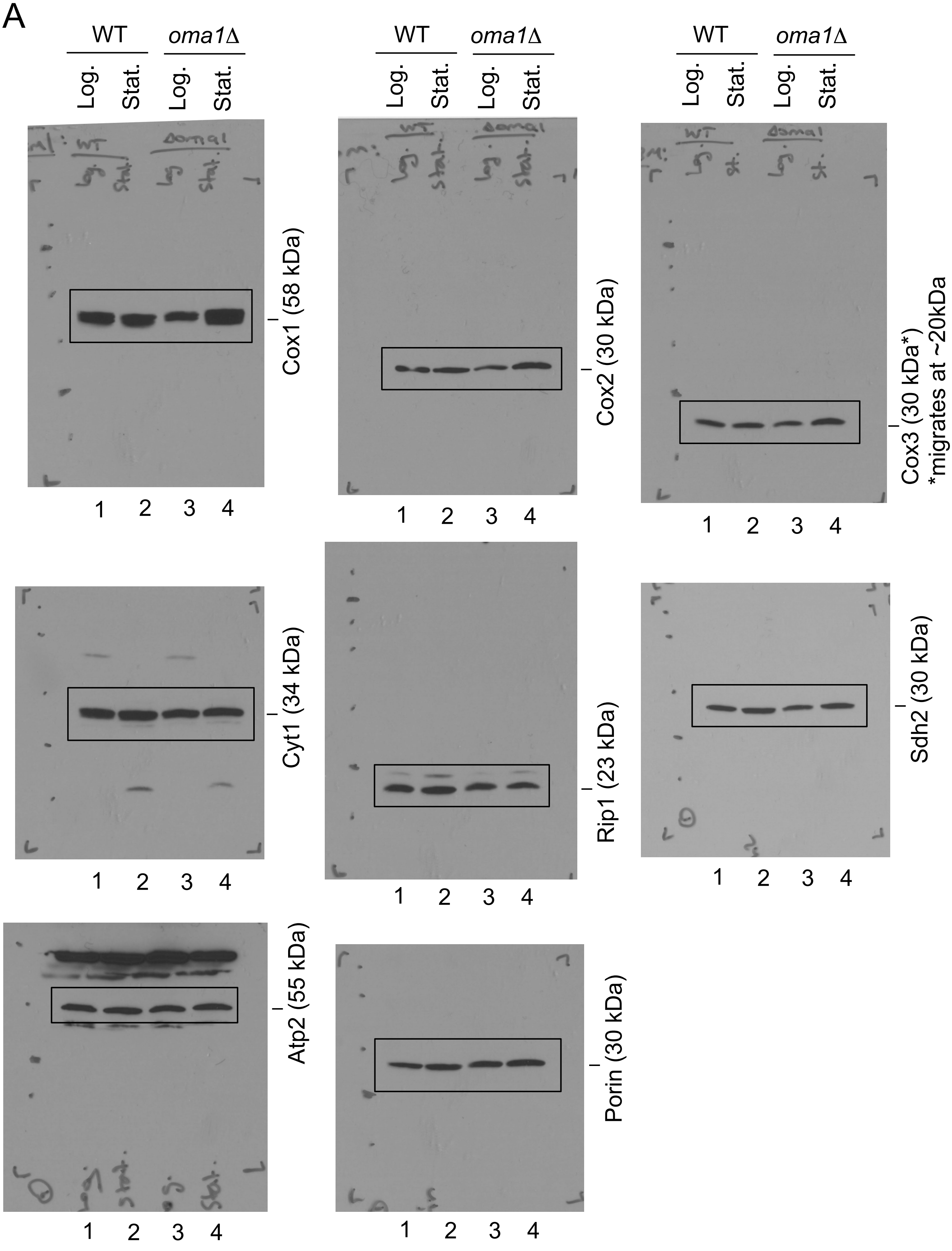


Bohovych, Figure S5

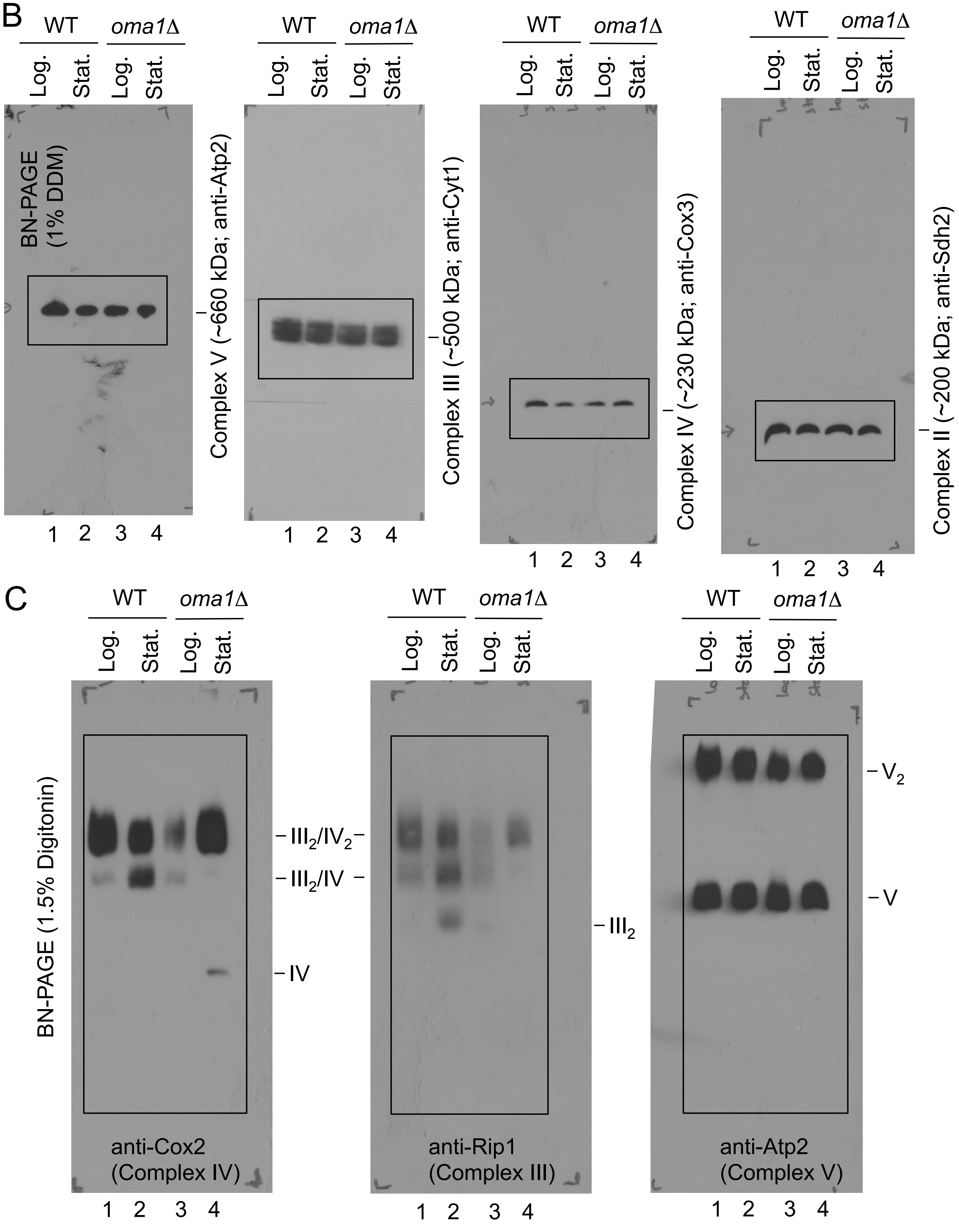


Bohovych, Figure S5 (continued)

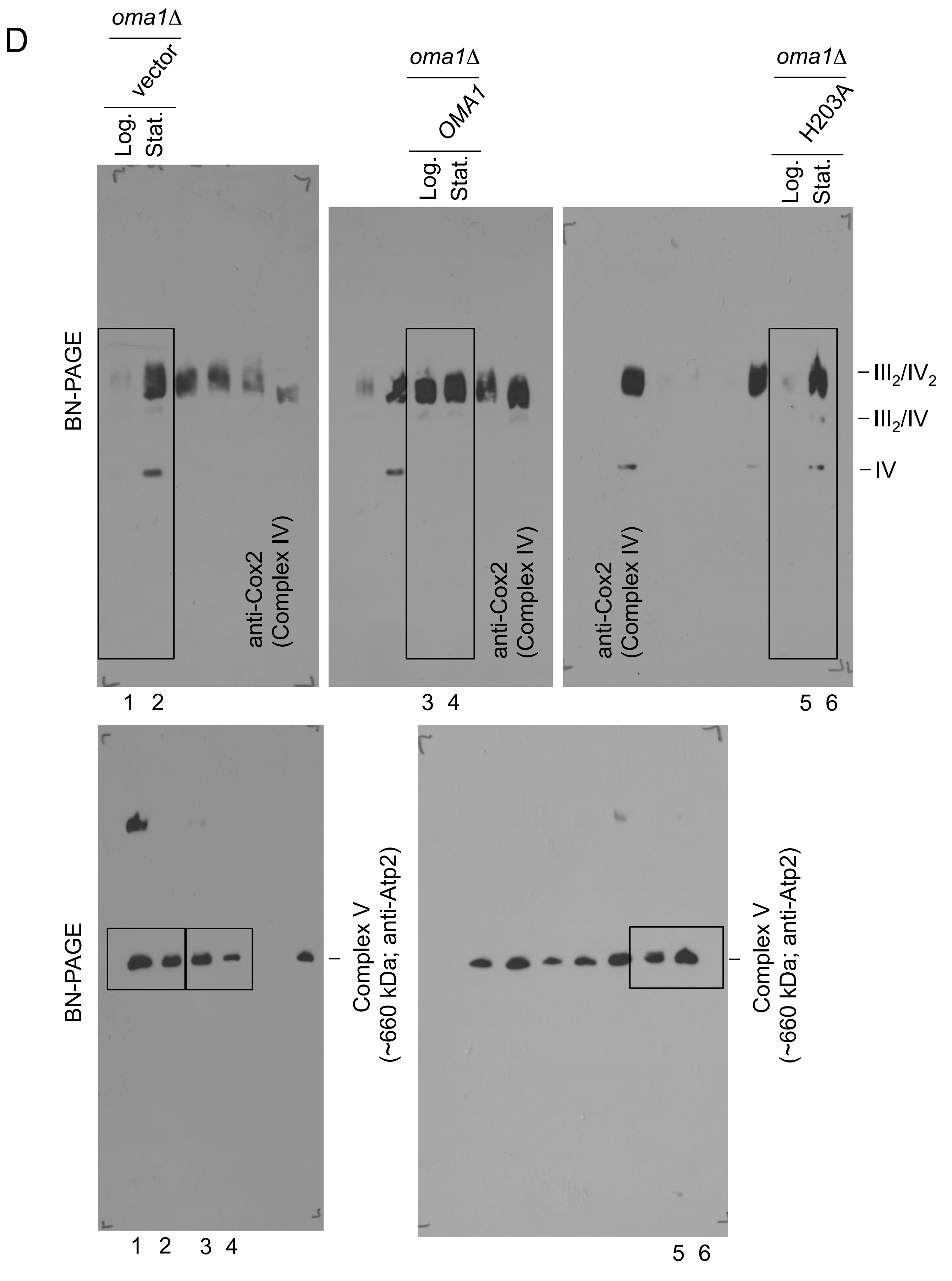


Bohovych, Figure S5 (continued)

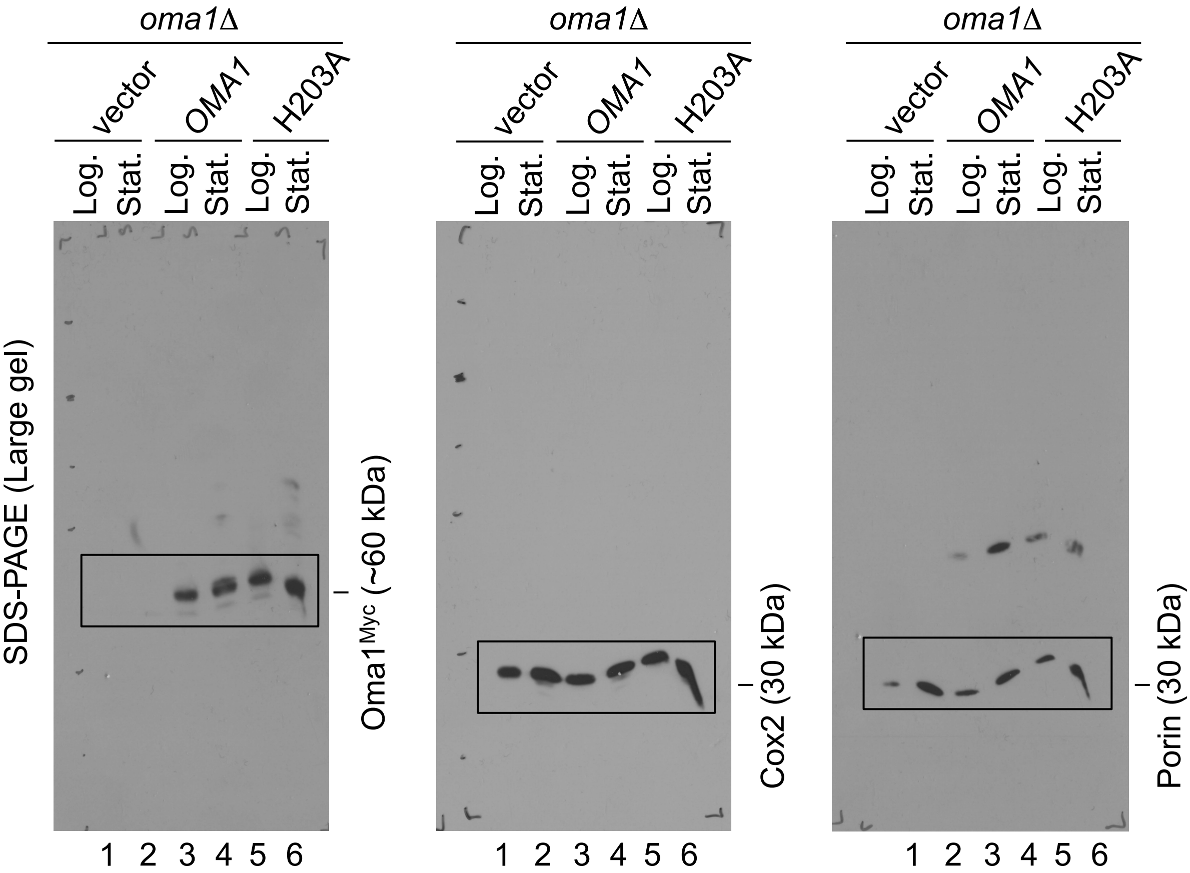


Bohovych, Figure S5 (continued)

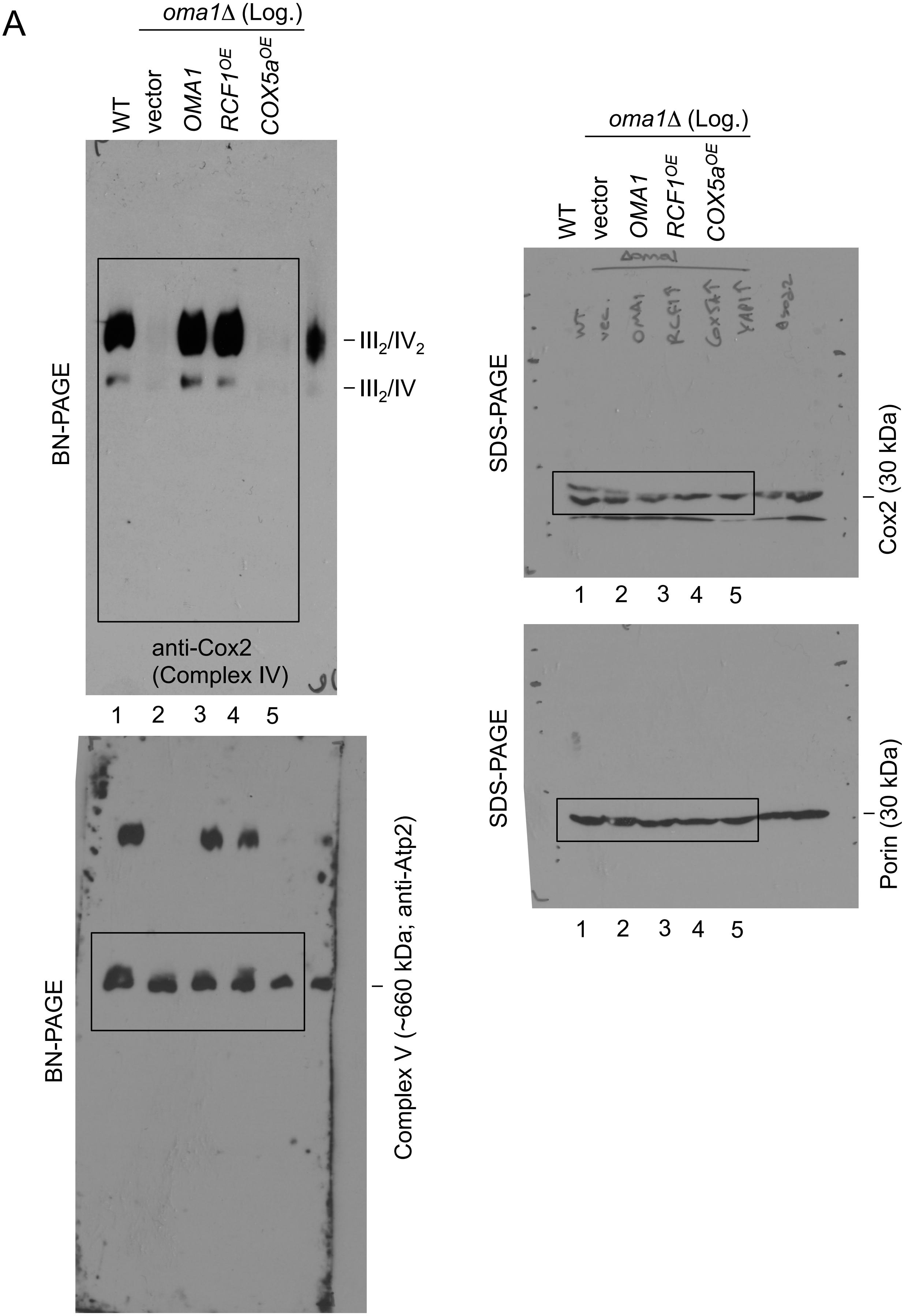


Bohovych, Figure S6

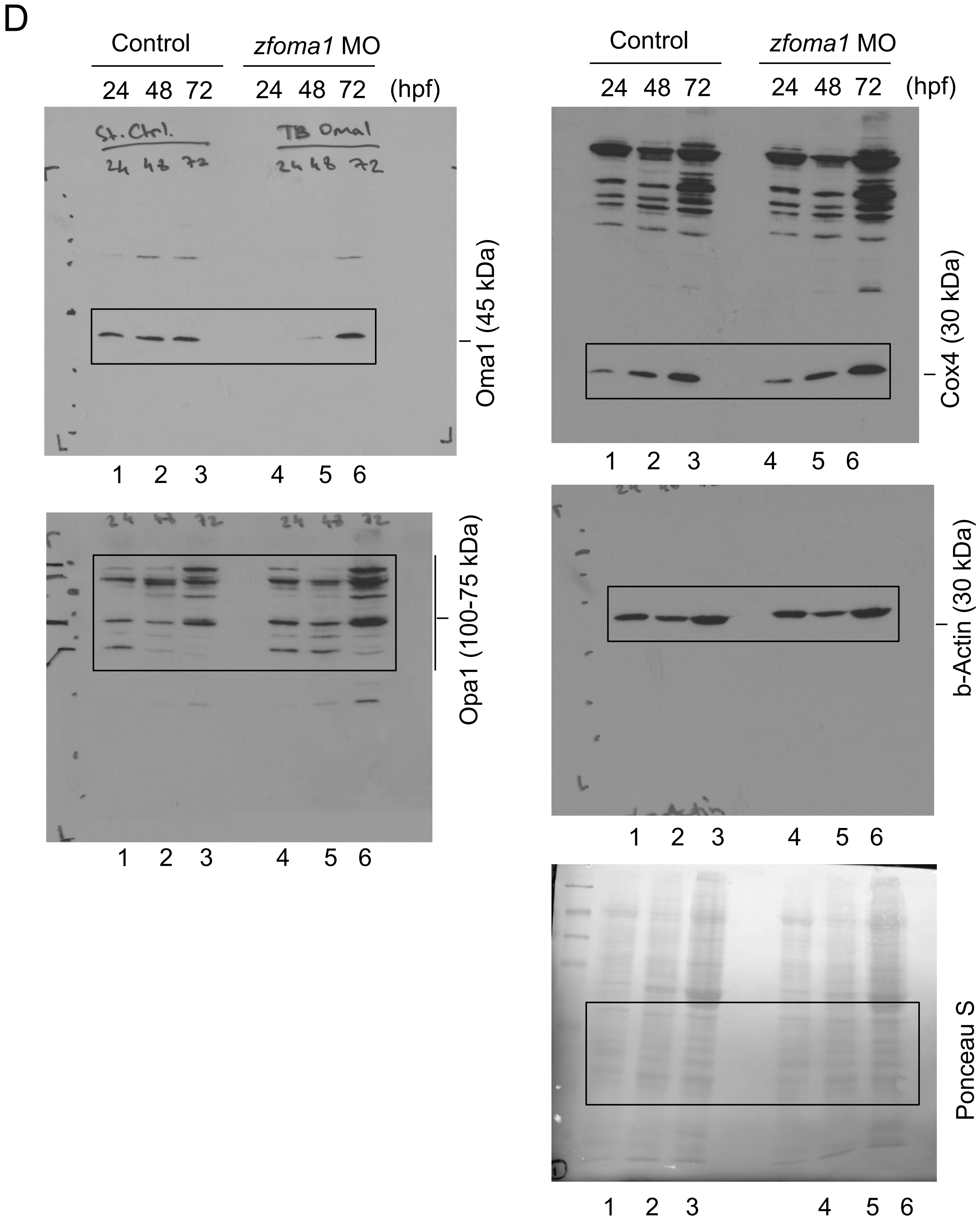


Bohovych, Figure S7

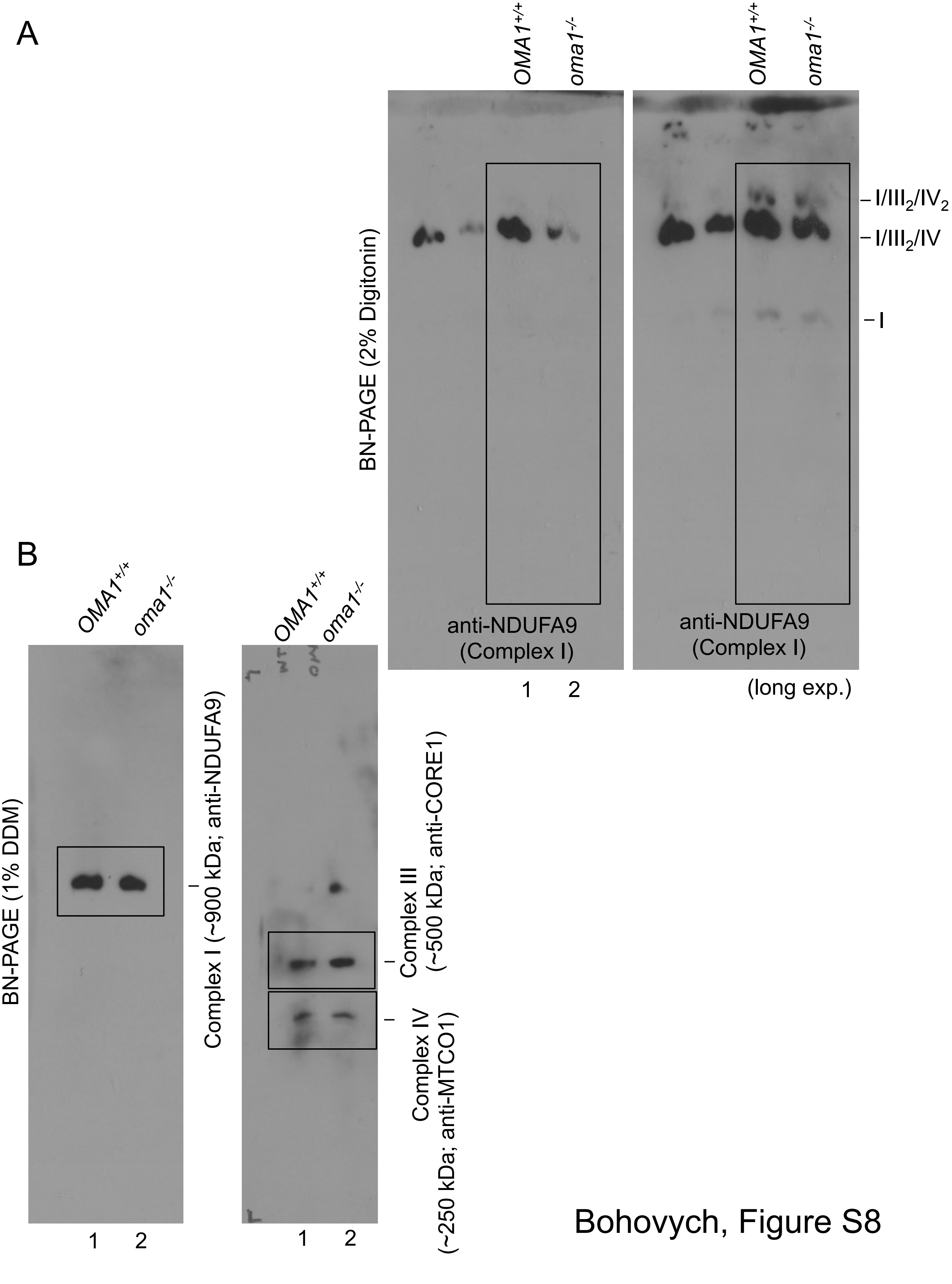


Bohovych, Figure S8

C

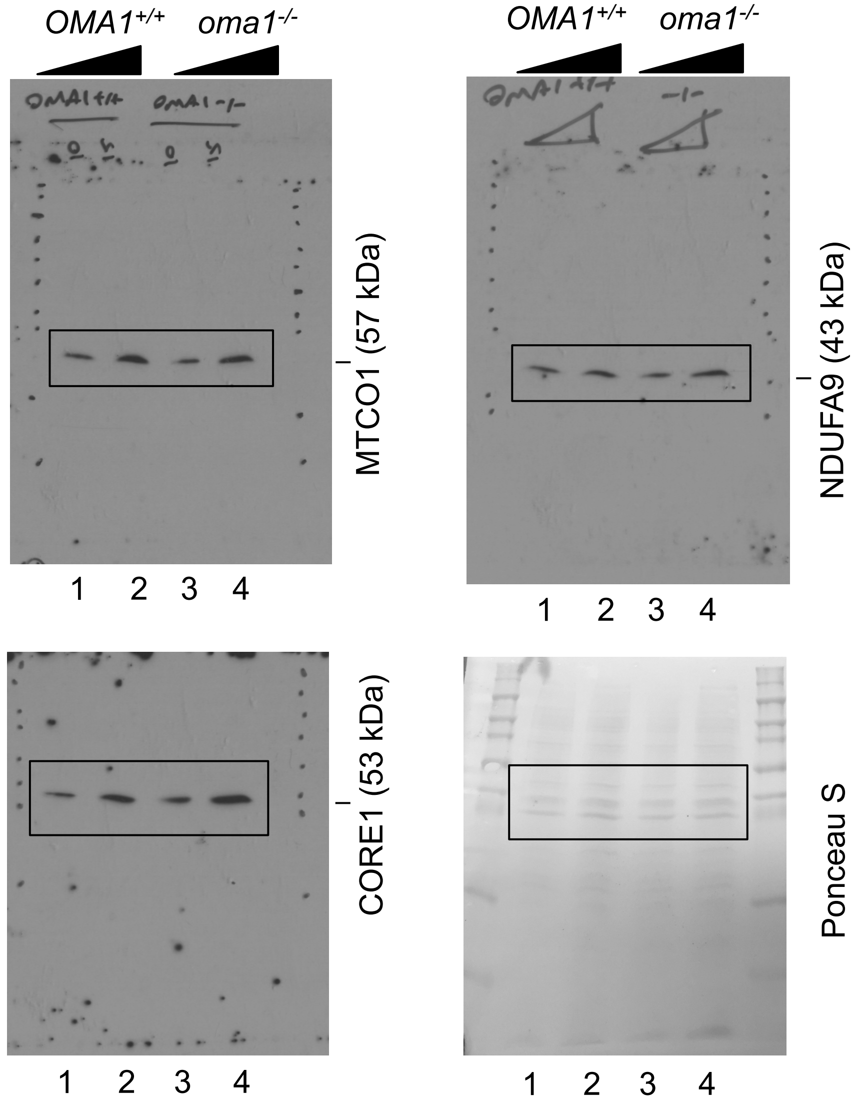


Bohovych, Figure S8 (continued)
